# High Dietary Sodium, Measured Using Spot Urine Samples, is Associated with Higher Blood Pressure among Young Adults in Haiti

**DOI:** 10.5334/gh.1187

**Published:** 2023-02-14

**Authors:** Adrienne Clermont, Vanessa Rouzier, Jean Lookens Pierre, Rodney Sufra, Eliezer Dade, Fabyola Preval, Stephano St-Preux, Marie Marcelle Deschamps, Alexandra Apollon, Kathryn Dupnik, Miranda Metz, Yanique Duffus, Shalom Sabwa, Lily D. Yan, Myung Hee Lee, Lawrence G. Palmer, Linda M. Gerber, Mark S. Pecker, Samuel J. Mann, Monika M. Safford, Daniel W. Fitzgerald, Jean W. Pape, Margaret L. McNairy

**Affiliations:** 1 Center for Global Health, Weill Cornell Medicine, 402 East 67th Street, New York, NY 10065, USA; 2 MD Program, Weill Cornell Medicine, 1300 York Avenue, New York, NY 10065, USA; 3 Haitian Group for the Study of Kaposi’s Sarcoma and Opportunistic Infections (GHESKIO), 33 Boulevard Harry Truman, Port-au-Prince 6110, Haiti; 4 Department of Physiology and Biophysics, Weill Cornell Medicine, 1300 York Avenue, New York, NY 10065, USA; 5 Department of Population Health Sciences, Weill Cornell Medicine, 402 East 67th Street, New York, NY 10065, USA; 6 Department of Medicine, Division of Nephrology and Hypertension, Weill Cornell Medicine, 1300 York Avenue, New York, NY 10065, USA

**Keywords:** Haiti, hypertension, salt consumption, spot urine samples

## Abstract

**Background:**

Hypertension (HTN) is the leading cardiovascular disease (CVD) risk factor in Haiti and is likely driven by poverty-related social and dietary factors. Salt consumption in Haiti is hypothesized to be high but has never been rigorously quantified.

**Methods:**

We used spot urine samples from a subset of participants in the population-based Haiti Cardiovascular Disease Cohort to estimate population mean daily sodium intake. We compared three previously validated formulas for estimating dietary sodium intake using urine sodium, urine creatinine, age, sex, height, and weight. We explored the association between dietary sodium intake and blood pressure, stratified by age group.

**Results:**

A total of 1,240 participants had spot urine samples. Median age was 38 years (range 18–93), and 48% were female. The mean dietary sodium intake was 3.5–5.0 g/day across the three estimation methods, with 94.2%–97.9% of participants consuming above the World Health Organization (WHO) recommended maximum of 2 g/day of sodium. Among young adults aged 18–29, increasing salt intake from the lowest quartile of consumption (<3.73 g/day) to the highest quartile (>5.88 g/day) was associated with a mean 8.71 mmHg higher systolic blood pressure (SBP) (95% confidence interval: 3.35, 14.07; p = 0.001). An association was not seen in older age groups. Among participants under age 40, those with SBP ≥120 mmHg consumed 0.5 g/day more sodium than those with SBP <120 mmHg (95% confidence interval: 0.08, 0.69; p = 0.012).

**Conclusions:**

Nine out of 10 Haitian adults in our study population consumed more than the WHO recommended maximum for daily sodium intake. In young adults, higher sodium consumption was associated with higher SBP. This represents an inflection point for increased HTN risk early in the life course and points to dietary salt intake as a potential modifiable risk factor for primordial and primary CVD prevention in young adults.

## Introduction

Cardiovascular disease (CVD) is the leading cause of adult mortality in Haiti, having surpassed HIV in the last decade [1,2]. Existing research suggests that hypertension (HTN) is the single most important risk factor for CVD in Haiti and may be driven by poverty-related social and dietary factors [3,4,5,6,7,8,9,10,11]. HTN also appears to occur earlier in Haiti, with a higher prevalence among young Haitian adults than similarly aged Black Americans [12,13]. In order to achieve the United Nations Sustainable Development Goal of a one-third reduction in CVD mortality by 2030, research is urgently needed to better understand the drivers of early-onset HTN in Haiti and develop interventions to improve health outcomes [14].

There is a well-established association between high dietary salt consumption and elevated blood pressure in both low- and high-income settings [15,16]. Salt consumption is hypothesized to be alarmingly high in Haiti – due to a number of factors including limited fresh food availability, cultural cooking practices, and use in food preservation – but this has yet to be rigorously quantified in a large-scale, population-based study [3,17,18]. Previous estimates based on small sample sizes and self-report surveys have been as high as 5–14 g/day of sodium [3,18]. A global modeling study based on survey data from other Caribbean countries estimated daily intake in Haiti to be 2.7 g/day of sodium [19]. All of these estimates exceed the World Health Organization (WHO) recommended maximum of 2 g/day of sodium (equivalent to 5 g/day of salt) [20].

Salt intake is notoriously difficult to quantify in self-report surveys, regardless of the setting. Most US cohorts rely on questionnaires that self-report frequency and amount of salt [21,22], or use nutritional methods such as 24-hour dietary recalls, food logs, or food frequency questionnaires to quantify salt intake, all of which have been found to correlate poorly with the ‘gold standard’ of 24-hour urine collection [23,24,25]. Although 24-hour urine collection is considered the most reliable method to measure sodium excretion, which reflects salt consumption, it is logistically difficult, with up to 50% not collected reliably even in resource-rich settings [26,27]. In a low-resource setting such as Haiti, accurate collection is even more challenging.

A single spot urine measurement is a practical alternative to a 24-hour urine collection to estimate population-level salt intake [28,29]. This logistically simpler approach has been implemented in both high- and low-income country settings [28,30,31,32,33], and a recent meta-analysis of 29 studies in 34 countries found spot urine measurements to be valid for estimating mean population salt intake [29]. In this meta-analysis, overall mean population sodium intake was 3.6 g/day estimated from spot urine, compared to 3.7 g/day measured from 24-hour urine. The spot urine estimate had high sensitivity (97%) and specificity (100%) for classifying mean population intake as above or below the WHO cutoff of 2 g/day of sodium.

This is the first large-scale quantitative estimate of dietary salt intake in Haiti. We used spot urine samples from participants in a cohort study of low-income, urban-dwelling adults to estimate average daily sodium intake. We hypothesized that the population mean intake would be greater than the WHO recommended maximum of 2 g/day of sodium, and that higher intake would be associated with higher blood pressure after controlling for demographic factors. We hypothesized that this would be true even in young adults, despite the common misconception that higher salt intake is only of concern in older adults or those with established hypertension.

## Methods

### Setting and Study Population

Haiti is a lower-middle-income country with a population of 11.4 million, including 2.6 million people living in the metropolitan area of Port-au-Prince, the capital city. This study was situated at GHESKIO (Groupe Haitien d’Etude du Sarcome de Kaposi et des Infections Opportunistes), a large public clinic and center for research and training located in downtown Port-au-Prince that primarily serves patients from nearby slum neighborhoods. GHESKIO was founded in 1982 to provide HIV care, and has since expanded to provide clinic- and community-based services for chronic diseases including HTN and other CVD.

The Haiti Cardiovascular Disease Cohort study (Clinicaltrials.gov registration number NCT03892265) is a population-based cohort study that aims to estimate the prevalence and incidence of CVD risk factors and events among adults in metropolitan Port-au-Prince [34]. The study cohort includes 3,005 adults aged 18 years or greater, enrolled in the study between March 2019 and August 2021. Participants were recruited using multistage random sampling based on national census blocks in selected areas of the city. By using a population-based sampling approach, rather than recruiting from GHESKIO’s patient population, the cohort is representative of low-income urban Haitian adults.

The study population eligible for this analysis was 1,560 participants who provided a non-fasting spot urine sample (those enrolled on or after August 6, 2020, when this sub-study was initiated and non-fasting urine sample collection began). Participants were excluded for the following reasons: 247 participants had missing urine samples, 62 participants reported diuretic use (known to affect urinary sodium excretion) [35], 10 participants had samples that could not be analyzed due to lab error, and one participant had missing demographic data. A sensitivity analysis including the 10 measurements with lab errors showed that their exclusion did not significantly alter our findings. A flowchart showing included and excluded participants is shown in Supplementary Figure 1.

### Measurements

This analysis uses cross-sectional data collected at the time of study enrollment. Participants completed a questionnaire including sociodemographic measures and health behaviors using validated questionnaires from the World Health Organization STEPwise Approach to Non-Communicable Disease Surveillance (WHO STEPS) methodology [36], as well as a clinical exam including medical history, anthropometric measurements, blood pressure measurements, and provision of blood and urine samples for laboratory testing.

Sociodemographic data included age, sex, education level, and income level. Education was categorized as having completed no education, primary, secondary, or higher than secondary education. Income was measured using categorical answer choices in Haitian currency, and then converted to US dollars (USD) for comparability (1 USD = roughly 90 Haitian gourdes during the study period). For the purposes of this analysis, income was dichotomized into two categories equivalent to ≤1 USD/day (including those reporting no income) and >1 USD/day.

Clinical exams were conducted by trained study nurses and physicians. Height and weight were measured using standardized techniques and equipment. Blood pressure measurements were taken according to WHO and American Heart Association (AHA) guidelines [36,37]. Blood pressure was assessed after a five-minute seated rest with both feet flat on the floor. Three consecutive blood pressure measurements were taken by a research nurse with an Omron HEM-907 series sphygmomanometer, which automatically took three blood pressure measurements separated by 60 seconds each. Systolic blood pressure (SBP) and diastolic blood pressure (DBP) for this analysis were calculated by taking the average of the second and third measurements. Blood pressure was categorized into normal (SBP <120 and DBP <80), pre-hypertension (SBP 120–139 or DBP 80–89), and hypertension (SBP ≥140 or DBP ≥90).

The study clinician also conducted a detailed review of the participant’s health conditions and medication usage, including self-report of dosage and frequency of all current CVD medications. Diuretic usage was defined as a participant taking either hydrochlorothiazide or furosemide at the time of study enrollment. Participants who reported currently taking non-diuretic anti-hypertensive medications were included in the hypertension category for analysis, regardless of measured blood pressure.

Finally, participants provided a 120 mL non-fasting spot urine sample. The study protocol specified that urine samples be collected 2–4 hours after a meal, which meant that most samples were collected in the late afternoon. This decision was based on expert consultation in order to best capture dietary sodium intake.

### Laboratory Analysis

Urine creatinine concentration was measured using a Vitros 350 Chemistry System (Ortho-Clinical Diagnostics, Raritan, NJ) or a DCA Vantage Analyzer (Siemens, Erlangen, Germany). Urine sodium concentration was measured using an Oakton Combination Ion-Sensitive Electrode for Sodium (Cole-Parmer, Vernon Hills, IL).

### Outcomes

Our primary outcome was estimated dietary sodium intake, which can be calculated based on 24-hour urinary sodium excretion. We compared three previously published methodologies for estimating 24-hour sodium excretion by using spot urine samples (formulas shown in Supplementary Table 1) [35,38,39,40].

The first two formulas are based on the principle that the ratio of sodium to creatinine in the spot urine sample is proportionate to the ratio excreted in 24 hours, so an estimated 24-hour creatinine excretion (based on various demographic factors) can be used to ‘scale up’ the results from the spot urine sample, as follows [35]:


Predicted\;{\rm{ }}24h{\rm{ }}\;Na = \;\frac{{Spot\;urine\;\left[ {Na} \right]}}{{Spot\;urine\;\left[ {Cr} \right]}}*Estimated\;24h{\rm{ }}\;Cr

The Kawasaki method was developed in 1993 based on a sample of 159 healthy Japanese adults (age 20–79 years) [38]. It uses sodium and creatinine concentrations from a second morning urine sample and estimates 24-hour creatinine excretion based on age, sex, height, and weight. The Tanaka method was developed in 2002 based on a sample of 591 Japanese adults (age 20–59 years) from the INTERSALT study (data collected in 1987–1988) [39]. It uses sodium and creatinine concentrations from a casual urine sample (i.e., time of day not specified) and estimates 24-hour creatinine excretion based on age, height, and weight.

The third formula uses a different methodological approach. The INTERSALT method was developed in 2013 based on a sample of 2,948 participants (age 20–59 years) from a variety of North American and European sites in the INTERSALT study (data collected in 1984–1987) [40]. Unlike the other formulas, INTERSALT is based on a regression analysis that uses urine sodium and creatinine concentrations from a casual urine sample, age, sex, and BMI as independent variables to predict 24-hour urine sodium excretion. This eliminates the step of estimating 24-hour urine creatinine excretion.

All three methods provide an estimate of 24-hour urine sodium *excretion*. Dietary sodium *intake* can be calculated by dividing the urine sodium excretion by 0.9, based on the assumption that 10% of sodium intake is lost through sweat and feces, and thus urinary excretion accounts for 90% of intake [41,42]. All results are reported here in terms of dietary *sodium* intake; if desired, dietary *salt* intake can be calculated by multiplying the dietary sodium intake by 2.5 [42].

After comparing the estimated population mean across all three estimation methods, we used the Kawasaki formula for subsequent analyses in this paper. This decision was based on a recent large-scale study suggesting that the Kawasaki formula may be most accurate in non-European populations [33].

### Statistical Analysis

Descriptive statistics for dietary sodium intake were calculated using the three estimation formulas. Correlations between the three methods were calculated using Pearson correlation coefficients.

Summary statistics for sodium intake were calculated for demographic subgroups using the Kawasaki method. Due to skewed distribution of sodium distribution, we used non-parametric tests to compare sodium intake between demographic subgroups. Sodium intake was compared between two groups (such as male vs. female) using a Wilcoxon rank-sum test, while sodium intake was compared between multiple categories (such as BMI categories) using the Kruskal-Wallis test.

Salt intake was split into quartiles and the association with systolic and diastolic blood pressure was explored within each age group. We used linear regression to determine systolic blood pressure by salt intake quartile, with age group added as a covariate. In order to investigate the differential association between salt and blood pressure by age group, interaction was allowed in the model. In order to measure the age-dependent salt and blood pressure association in a clinically relevant way, we compared salt intake for participants with systolic blood pressure ≥120 mmHg vs. <120 mmHg, separately for age <40 years and age ≥40 years, using the Wilcoxon rank-sum test.

Data were analyzed using R version 4.0.2.

### Ethics Approval

This study was approved by the institutional review boards at Weill Cornell Medicine and GHESKIO (IRB #1803019037). Prior to study implementation, meetings were held with community, school, and religious leaders, as well as GHESKIO’s Community Advisory Board, to answer questions regarding the study. Written informed consent was obtained for all participants prior to enrollment.

## Results

Out of a total of 3,005 participants in the Haiti Cardiovascular Disease Cohort, 1,560 participants were eligible for the urine sodium analysis based on their enrollment dates. Among these participants, 1,240 had complete data to include in the analysis (79.5%). A comparison of baseline characteristics for the overall Haiti Cardiovascular Disease Cohort and those included in this analysis is in Supplementary Table 2.

Baseline characteristics of the study participants are shown in Table 1. The study population was 52.4% male and had a median age of 38 years (IQR 26–53; range 18–93). The majority of participants (65.4%) had a secondary education or higher. Most participants (70.1%) made ≤1 USD/day, including 65.2% who reported earning no formal income. The median BMI was 23 kg/m^2^ (IQR: 21–27). About half of participants (51.3%) had a normal BMI, while 6.5% were in the underweight category and 42.2% were in the overweight and obese categories. There were 53.9% of participants in the normal blood pressure range, 22.8% in the pre-hypertension range, and 23.3% in the hypertension range. This latter category included a small number of participants (n = 30; 2.4% of study population) who were on anti-hypertensive medications.

**Table 1:** Characteristics of study participants.


	PARTICIPANTS

Participants	1,240

**Sex**	

Male	650 (52.4%)

Female	590 (47.6%)

**Age (years)**	

Median (IQR, range)	38 (26–53, 18–93)

18–29	427 (34.4%)

30–39	228 (18.4%)

40–49	189 (15.2%)

50–59	199 (16.1%)

≥60	197 (15.9%)

**Education**	

None	175 (14.1%)

Primary	254 (20.5%)

Secondary	610 (49.2%)

Higher than secondary	201 (16.2%)

**Income**	

≤1 USD/day	869 (70.1%)

>1 USD/day	371 (29.9%)

**BMI (kg/m^2^)**	

Median (IQR)	23 (21–27)

Underweight (<18.5)	80 (6.5%)

Normal (18.5–24.9)	636 (51.3%)

Overweight (25–29.9)	324 (26.1%)

Obese (≥30.0)	200 (16.1%)

**Blood pressure (mmHg)**	

Median (IQR, range) – SBP	117 (107–134, 80–240)

Median (IQR, range) – DBP	70 (61–82, 38–147)

Normal blood pressure (SBP <120 and DBP <80)	668 (53.9%)

Pre-hypertension (SBP 120–139 or DBP 80–89)	283 (22.8%)

Hypertension (SBP ≥140 or DBP ≥90 or on medication)	289 (23.3%)


*Note*: Categories may not sum to 100% due to rounding. IQR = interquartile range, USD = United States dollar, BMI = body-mass index, SBP = systolic blood pressure, DBP = diastolic blood pressure.

### Population mean dietary sodium intake

The population mean dietary sodium intake, calculated using the three estimation methods, is shown in Table 2. The correlation between the three methods was high, with pairwise correlations ranging from 0.74–0.96 (Supplementary Figure 2). The estimated population mean ranged from a low of 3.5 g/day of sodium using the INTERSALT formula (95% confidence interval [CI]: 3.4, 3.5) to a high of 5.0 g/day of sodium using the Kawasaki formula (95% CI: 4.8, 5.1). The proportion of the population above the WHO recommended maximum of 2 g/day of sodium ranged from 94.2% to 97.9%, depending on the estimation method.

**Table 2:** Estimated dietary sodium intake (grams/day) using three validated methods.


	MEAN (95% CI)	% OF POPULATION ABOVE 2 g/DAY OF SODIUM*

Kawasaki method	5.0 (4.8, 5.1)	97.9%

Tanaka method	3.8 (3.7, 3.8)	97.7%

INTERSALT method	3.5 (3.4, 3.5)	94.2%


* World Health Organization recommended maximum daily intake. CI = confidence interval.

### Mean dietary sodium intake among subgroups

The mean dietary sodium intake by sociodemographic subgroup is shown in Table 3 using the Kawasaki formula. There was a statistically significant difference in sodium intake by sex, with men consuming more than women. This was not due solely to differences in body mass; sodium consumed per kilogram of body weight was higher among men than women (Supplementary Figure 3). There was a statistically significant positive relationship between education level and sodium intake, while there was a statistically significant negative relationship between age and sodium intake, and between BMI and sodium intake.

**Table 3:** Estimated dietary sodium intake (grams/day), by demographic group.


DEMOGRAPHIC GROUP	MEAN (95% CI)	*P*-VALUE*

**Sex**		<0.001

Male	5.4 (5.2, 5.6)	

Female	4.5 (4.3, 4.6)	

**Age (years)**		<0.001

18–29	5.4 (5.3, 5.6)	

30–39	5.0 (4.8, 5.2)	

40–49	4.8 (4.5, 5.0)	

50–59	4.7 (4.5, 5.0)	

≥60	4.3 (3.9, 4.6)	

**Education**		<0.001

None	4.4 (4.1, 4.7)	

Primary	4.7 (4.4, 5.0)	

Secondary	5.1 (4.9, 5.2)	

Higher than secondary	5.3 (5.1, 5.5)	

**Income**		0.16

≤1 USD/day	5.0 (4.9, 5.1)	

>1 USD/day	4.8 (4.6, 5.0)	

**BMI (kg/m^2^)**		<0.001

Underweight (<18.5)	5.0 (4.7, 5.3)	

Normal (18.5–24.9)	5.0 (4.9, 5.2)	

Overweight (25–29.9)	5.1 (4.8, 5.3)	

Obese (≥30.0)	4.4 (4.2, 4.7)	


* P-value based on Wilcoxon rank-sum test for comparisons between two groups and Kruskal-Wallis test for comparisons between three or more groups.

### Association between dietary sodium intake and blood pressure

**Figure 1:**
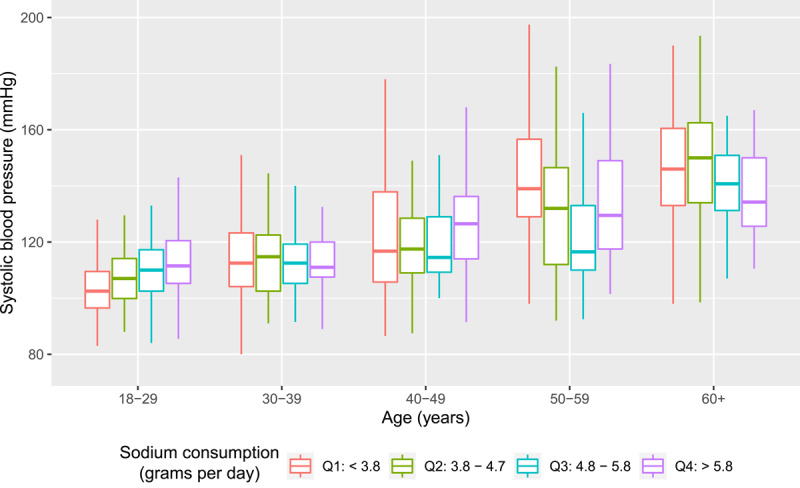
Systolic blood pressure by sodium consumption quartile, stratified by age group.

The relationship between sodium intake (divided into quartiles) and systolic blood pressure, by age group, is shown in Figure 1 using the Kawasaki method. Age was found to be a significant confounder in the relationship between sodium intake and blood pressure; as a result, our analyses are stratified by age group. There was a statistically significant positive association between sodium intake and systolic blood pressure within the age <30 years group: Sodium intake in the top consumption quartile (>5.8 g/day) compared to the bottom quartile (<3.8 g/day) was associated with 8.71 mmHg (95% CI: 3.35, 14.07; p = 0.001) higher systolic pressure. There was also a statistically significant difference in SBP between the bottom quartile and the second quartile (6.19 mmHg, p = 0.029), while the difference in SBP between the bottom quartile and third quartile was not significant (4.71 mmHg, p = 0.114).

The relationship was not statistically significant within the other age groups. There was no statistically significant relationship between sodium consumption quartile and diastolic blood pressure in any age group.

**Figure 2:**
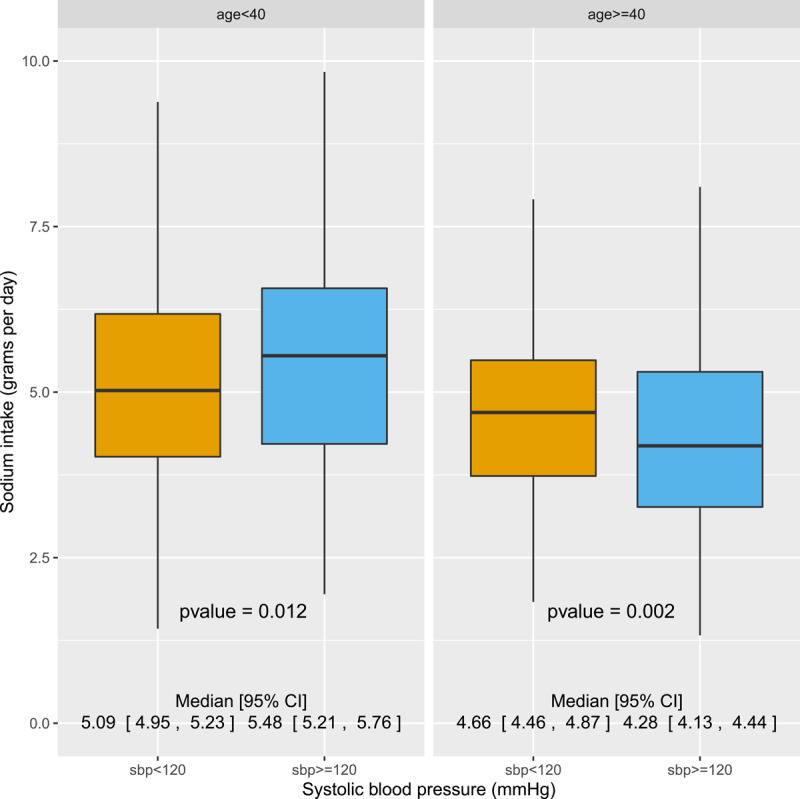
Sodium intake comparison between systolic blood pressure groups, stratified by age group.

In order to further describe the relationship between age, sodium consumption and blood pressure, we dichotomized the participants into age <40 years and age ≥40 years (the age cutoff at which the AHA recommends beginning CVD screening) and compared the dietary sodium intake between participants with SBP ≥120 mmHg and SBP <120 mmHg (the cutoff for normal SBP) (Figure 2). There was a statistically significant *positive* relationship between SBP and salt intake among participants under age 40, with those in the SBP ≥120 mmHg category consuming 0.5 g/day more sodium than those in the SBP <120 mmHg category (95% CI: 0.08, 0.69; p = 0.012). There was a statistically significant *negative* relationship among participants over age 40, with those in the SBP ≥120 mmHg category consuming 0.5 g/day less sodium than those in the SBP <120 mmHg category (95% CI: 0.15, 0.66; p = 0.002).

## Discussion

The results of our analysis show that salt consumption among urban adults in Haiti is high, with the vast majority (94.2–97.9%) of participants consuming more than the WHO recommended maximum of 2 g/day of sodium. Among young people aged 18–29 years, increasing salt intake from the lowest quartile of consumption (<3.8 g/day) to the highest quartile (>5.8 g/day) was associated with an 8.71 mmHg higher SBP. This finding has important implications for health outcomes, as previous research has shown that reductions as small as 5 mmHg are associated with decreased CVD events and mortality in adults, across the range of SBP values [43].

While the average daily sodium intake for our study population was high, estimated at 3.5–5.0 g/day, it is less than previously estimated in small studies from Haiti relying on self-report, which hypothesized rates as high as 5–14 g/day [3,18]. Ethnographic research in Haiti attributes high consumption to a number of cultural factors including taste preferences, folk beliefs regarding the positive properties of salt, prevalent use of salty seasonings such as bouillon cubes, and the need to preserve food in the absence of refrigeration [17,18]. It is important to note that excessive salt consumption is not unique to Haiti. The Global Burden of Disease study found that nearly every country (99.2% of the global population) exceeds the WHO recommended maximum. Their model estimated a worldwide average of 3.95 g/day of sodium, including as high as 5.51 g/day in Central Asia [19]. However, they estimated an average of 2.61 g/day for the Caribbean, suggesting Haiti may be a high outlier for its region.

Our study found significant variations in dietary sodium intake by sociodemographic group, with higher levels among men and younger age groups. These associations align with self-reported dietary data from our study cohort, which showed that young males were more likely to report a number of dietary risk factors, including consuming fried foods and sugar-sweetened beverages, purchasing meals from street vendors, and frequently adding salt to meals cooked at home [44]. It is also in line with previous research from China that found greater salt consumption among men compared to women and younger adults compared to older adults [45], as well as a large-scale UK Biobank study that found participants in the highest quintiles of sodium excretion were younger and more likely to be men [46]. We also found higher dietary sodium intake among higher education level groups, which may be due to increased access to processed foods and meals cooked outside the home. There was no significant relationship between income and sodium consumption, although our income data only captures formal income and thus may not accurately capture the household purchasing power of our participants.

Among participants under age 40, there was a statistically significant positive association between blood pressure and sodium intake, with participants with SBP <120 mmHg consuming 0.5 g/day less of sodium compared to participants with SBP ≥120 mmHg. This is in line with several previous studies that have found a positive relationship between blood pressure and sodium intake using spot urine samples [33,46]. This finding identifies excessive salt consumption as a potential modifiable risk factor for early-onset HTN in Haiti, which takes place at higher rates and at a younger age than in the United States [12,13].

In contrast, among participants over age 40, there was a statistically significant negative association, meaning that participants with SBP <120 mmHg consumed 0.5 g/day more of sodium compared to participants with SBP ≥120 mmHg. The reason for this relationship is not clear with cross-sectional data alone. One possible explanation is survivorship bias, that is, individuals with high salt consumption and high blood pressure had increased premature mortality and thus could not be included in our study. Or it may be that some older patients had previously been diagnosed by a healthcare provider with HTN and are making an effort to decrease their salt consumption as a result. It is unlikely that anti-hypertensive medication use is an explanatory factor, as only 30 participants (2.4% of the study population) were taking these medications. Prospective data are needed to establish a causal link.

### Strengths and Limitations

Our study is the first large-scale quantification of sodium intake in Haiti using a population-based cohort. By rigorously following WHO and AHA guidelines for taking blood pressures, our measurements are comparable to US and international cohorts. Another strength of our study is the use of three previously validated methods for estimating 24-hour sodium excretion based on a spot urine sample. The use of multiple methods, all yielding similar results, allows for higher confidence in this initial quantification of spot urine sodium in Haiti. To our knowledge, this is the first large-scale study to use spot urine sodium estimation in the Caribbean region.

Our results may underestimate true sodium intake in Haiti. In this study, we assume that 90% of sodium intake is excreted in the urine, while the remaining 10% are lost in sweat and feces. Previous data suggest that vigorous physical activity and climate (heat and humidity) may lead to a higher rate of sodium loss in sweat [42]. This is likely the case in Haiti, which has a tropical climate.

The participants included in this analysis differed from those in the overall population-based Haiti Cardiovascular Disease Cohort (see Supplementary Table 2). Our analysis included more men and fewer participants with hypertension than the overall study population. While this may limit generalizability to the Haitian population at large, this is the first study to provide quantitative data on sodium consumption in Haiti. Our findings are also limited by the fact that we were not able to account for all possible confounders in the relationship between sodium intake and blood pressure. There are a number of socioeconomic and biomedical factors that we were not able to measure or control for in our analysis. One area for future research would be to examine potassium consumption among Haitians, as this has been shown in previous studies to have an impact on CVD risk [47].

### Conclusions

Modeling studies suggest that nearly one in 10 CVD deaths worldwide can be attributed to excess salt intake [48]. In this large-scale population-based cohort of urban Haitian adults, 9 out of every 10 participants consumed more sodium than the WHO recommended maximum.

We found the expected positive relationship between urine sodium and blood pressure in younger but not older adults, suggesting that a follow-up study with 24-hour urines is warranted. Among young adults in particular, higher sodium consumption was associated with higher SBP. This represents an important inflection point for increased HTN risk early in the life course and points to the modifiable risk factor of dietary salt intake as a potential pathway for primordial and primary CVD prevention in young adults.

Further research is needed to better understand knowledge and attitudes surrounding salt use, and what types of dietary modifications could reduce salt consumption. This type of formative research, taking into account culture-specific factors, will help to better inform future public health interventions and is urgently needed to meet global targets for reduction in CVD and improve the health outcomes of Haitians.

## Data accessibility statement

Data contain potentially identifying and sensitive patient information. Deidentified data used for this analysis are available upon request after signing a data access and use agreement, gaining a provision of approval by the GHESKIO ethics board, and demonstrating that the external investigative team is qualified and has documented evidence of human research protection training. Requests may be addressed to the corresponding author.

## Additional File

The additional file for this article can be found as follows:

10.5334/gh.1187.s1 Supplemental File. Supplementary Figures 1 to 3 and Supplementary Tables 1 and 2.
