# Supplementary material for: High Dietary Sodium, Measured Using Spot Urine Samples, is Associated with Higher Blood Pressure among Young Adults in Haiti
Source: Glob Heart. 2023 Feb 14;18(1):5. doi: 10.5334/gh.1187 (PMC9936908; doi:10.5334/gh.1187)
Supplement: Supplemental File. — Supplementary Figures 1 to 3 and Supplementary Tables 1 and 2. [file gh-18-1-1187-s1.pdf]

Supplementary Figure 1: Inclusion and exclusion criteria for Haiti Cardiovascular Disease (CVD) Cohort participants in the urine sodium sub-study

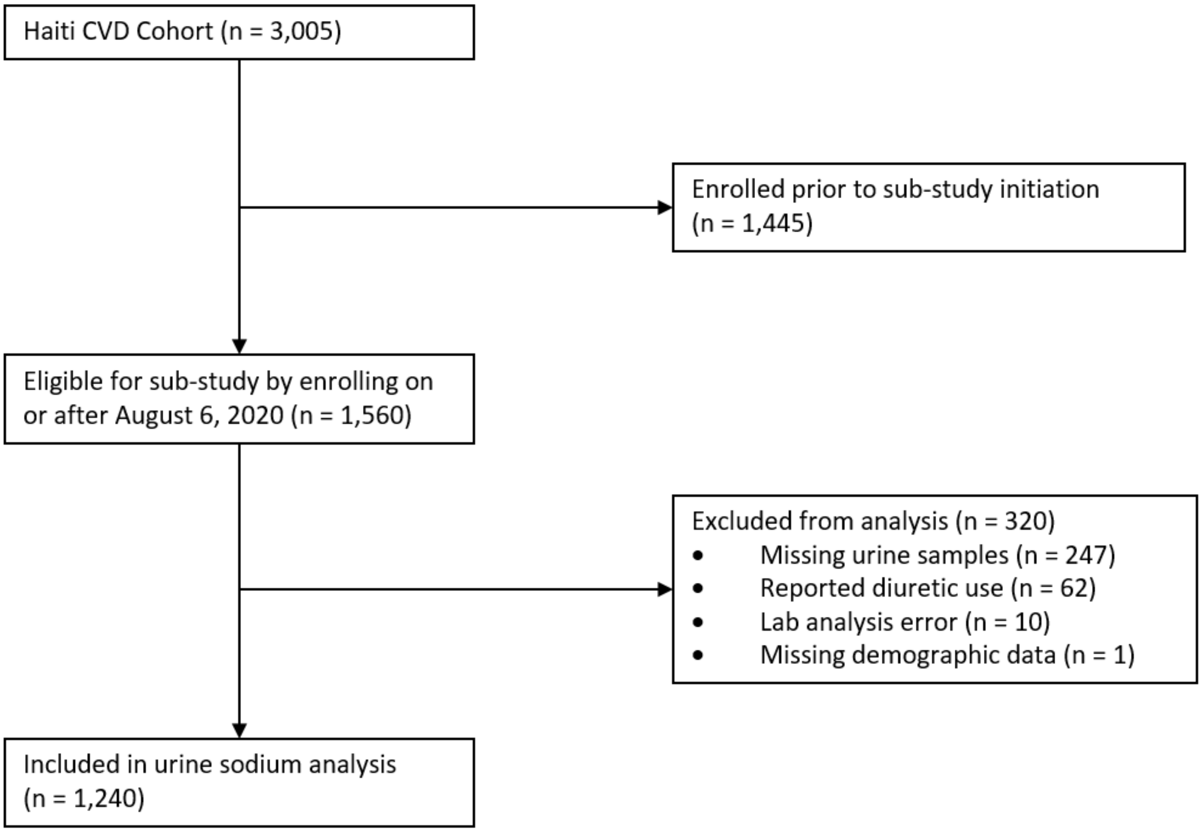

Supplementary Figure 2: Distribution and correlation between three methods of estimating dietary sodium intake for study participants

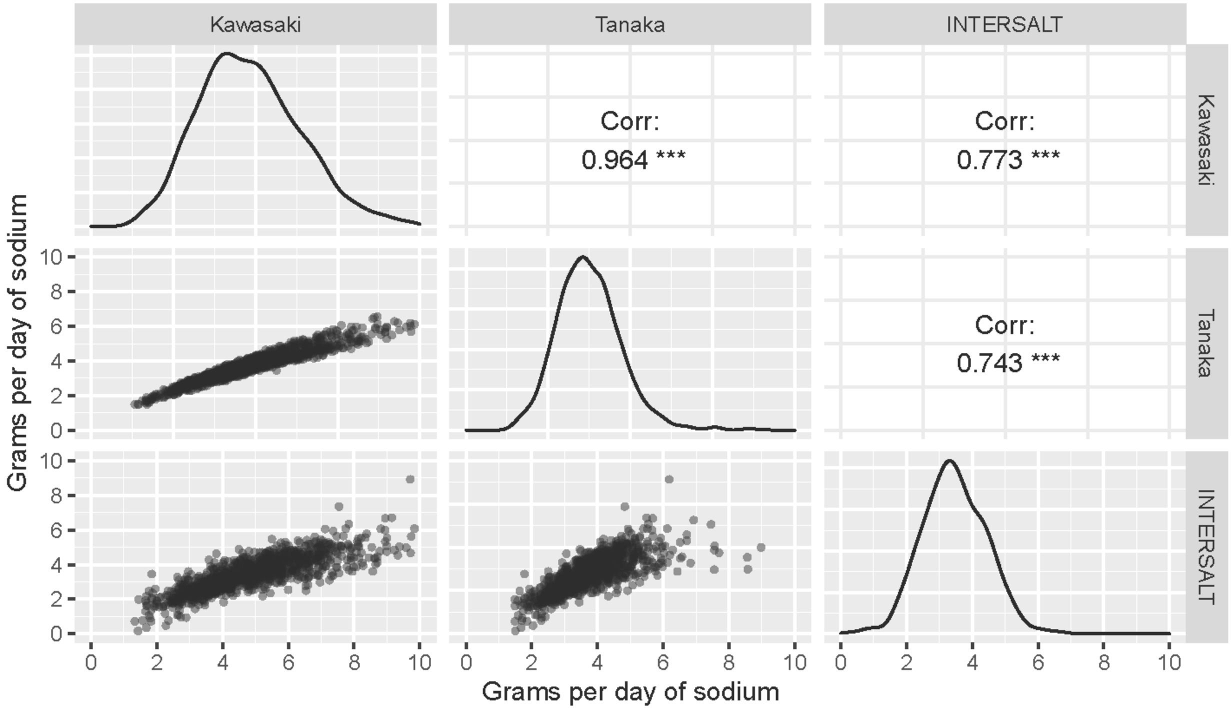

*Distributions of sodium intake using each estimation method are shown in diagonal panels. Scatter plots of pairs of estimation methods are shown in lower non-diagonal panels and the pairwise correlation coefficients are displayed in upper non-diagonal panels. All pairwise correlation coefficients are statistically significant with p <0.001, denoted by ***.*

Supplementary Figure 3: Average daily consumption of sodium per kilogram of body weight, by sex

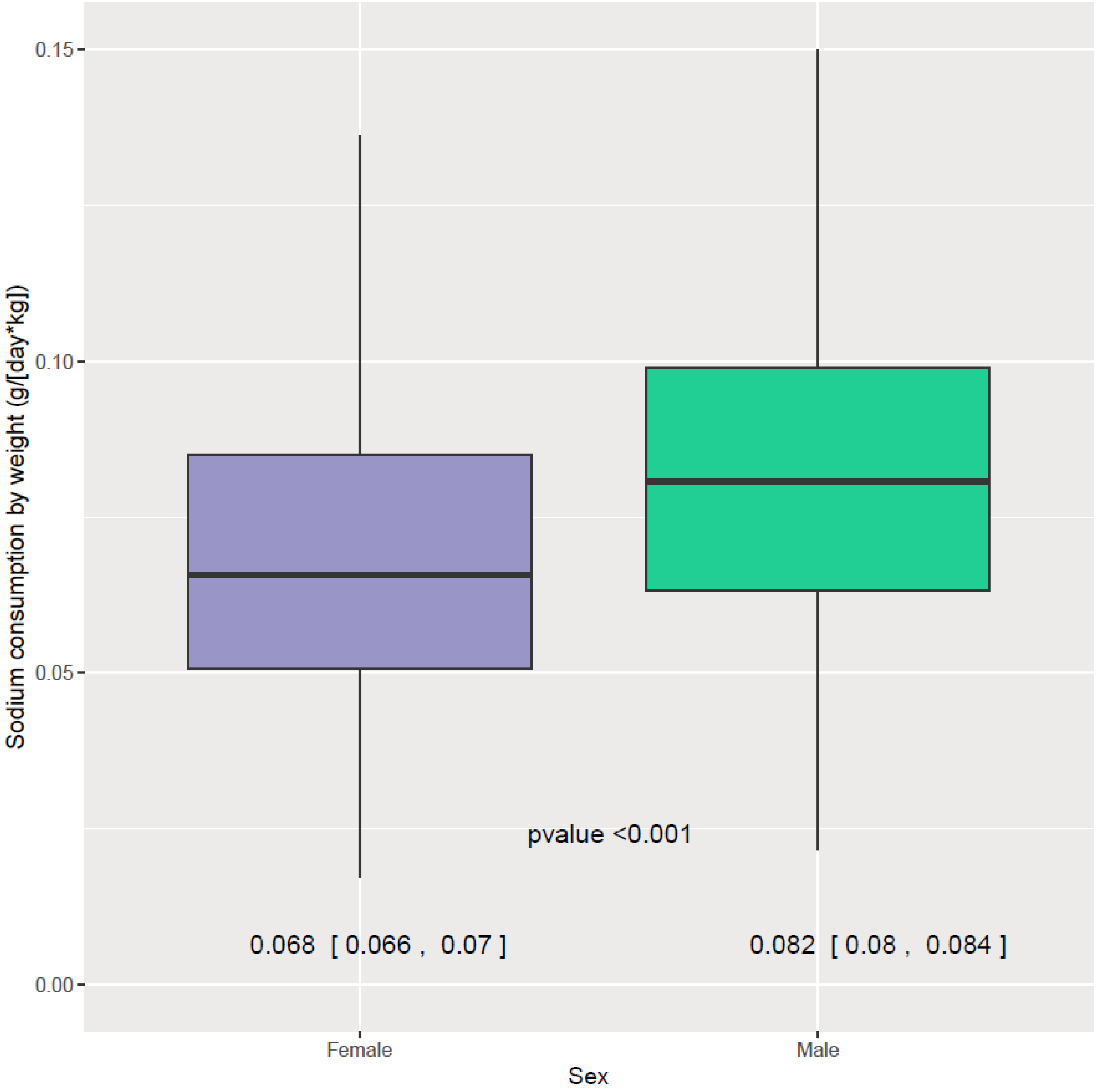

Values shown in figure: Median [95% confidence interval]

Supplementary Table 1: Published formulas for estimating 24-hour sodium excretion from spot urine sodium samples

Method	Formula for 24-hour sodium excretion	Description
Kawasaki method[1]	*Male:* 23 ÷ 1000 × 16.3 × {spot Na (mmol/L) ÷ [spot Cr (mg/dL) × 10] × [-12.63 × age (years) + 15.12 × weight (kg) + 7.39 × height (cm) – 79.9]}$^{0.5}$<br><br>*Female:* 23 ÷ 1000 × 16.3 × {spot Na (mmol/L) ÷ [spot Cr (mg/dL) × 10] × [-4.72 × age (years) + 8.58 × weight (kg) + 5.09 × height (cm) – 74.5]}$^{0.5}$	• Derived in Japanese population<br>• Second morning urine
Tanaka method[2]	23 ÷ 1000 × 21.98 × {spot Na (mmol/L) ÷ [spot Cr (mg/dL) × 10] × [-2.04 × age (years) + 14.89 × weight (kg) + 16.14 × height (cm) – 2244.45]}$^{0.392}$	• Derived in Japanese population<br>• Casual urine sample (i.e., time not specified)
INTERSALT method[3]	*Male:* 23 ÷ 1000 × [23.51 + 0.45 × spot Na (mmol/L) – 3.09 × spot Cr (mmol/L) + 4.16 × BMI (kg/m^2) + 0.22 × age (years)]<br><br>*Female:* 23 ÷ 1000 × {3.74 + 0.33 × spot Na (mmol/L) – 2.44 × spot Cr (mmol/L) + 2.42 × BMI (kg/m^2) + 2.34 × age (years) + 0.03 × [age (years)]^2}	• Derived in North American and European population<br>• Casual urine sample (i.e., time not specified)

References

1 Kawasaki T, Itoh K, Uezono K, Sasaki H. A simple method for estimating 24 h urinary sodium and potassium excretion from second morning voiding urine specimen in adults. *Clin Exp Pharmacol Physiol* 1993; **20**: 7–14.

2 Tanaka T, Okamura T, Miura K, *et al.* A simple method to estimate populational 24-h urinary sodium and potassium excretion using a casual urine specimen. *J Hum Hypertens* 2002; **16**: 97–103.

3 Brown IJ, Dyer AR, Chan Q, *et al.* Estimating 24-hour urinary sodium excretion from casual urinary sodium concentrations in Western populations: the INTERSALT study. *Am J Epidemiol* 2013; **177**: 1180–92.

Supplementary Table 2: Characteristics of study participants included in the dietary sodium analysis compared to the overall Haiti CVD Cohort

	**Salt Study Analysis Population**	**Overall Haiti CVD Cohort**
Participants	1,240	3,005
Sex		
Male	650 (52.4%)	1,260 (41.9%)
Female	590 (47.9%)	1,745 (58.1%)
Age (years)		
Median (IQR, range)	38 (26-53, 18-93)	40 (27-55, 18-93)
18-29	427 (34.4%)	890 (29.6%)
30-39	228 (18.4%)	569 (18.9%)
40-49	189 (15.2%)	533 (17.7%)
50-59	199 (16.1%)	499 (16.6%)
≥60	197 (15.9%)	514 (17.1%)
Education		
None	175 (14.1%)	427 (14.2%)
Primary	254 (20.5%)	646 (21.5%)
Secondary	610 (49.2%)	1,478 (49.2%)
Higher than secondary	201 (16.2%)	445 (14.8%)
Income		
≤1 USD/day	869 (70.1%)	2,105 (70.0%)
>1 USD/day	371 (29.9%)	891 (29.7%)
BMI (kg/m^2)		
Median (IQR, range)	23 (21-27, 12-61)	24 (21-28, 12-62)
Underweight (<18.5)	80 (6.5%)	144 (4.8%)
Normal (18.5-24.9)	636 (51.3%)	1,557 (51.8%)
Overweight (25-29.9)	324 (26.1%)	784 (26.1%)
Obese (≥30.0)	200 (16.1%)	515 (17.1%)
Blood pressure (mmHg)		
Median (IQR, range) – SBP	117 (107-134, 80-240)	119 (107-138, 71-240)
Median (IQR, range) – DBP	70 (61-82, 38-147)	72 (63-84, 38-147)
Normal blood pressure (SBP<120 and DBP<80)	668 (53.9%)	1,470 (48.9%)
Pre-hypertension (SBP 120-139 or DBP 80-89)	283 (22.8%)	640 (21.3%)
Hypertension (SBP≥140 or DBP≥90, or on HTN medications)	289 (23.3%)	871 (29.0%)

Note: Categories may not sum to 100% due to rounding
CVD = cardiovascular disease, IQR = interquartile range, USD = United States dollar, BMI = body-mass index, SBP = systolic blood pressure, DBP = diastolic blood pressure, HTN = hypertension
